# Clinical Presentation of Klinefelter's Syndrome: Differences According to Age

**DOI:** 10.1155/2012/324835

**Published:** 2012-01-12

**Authors:** Néstor Pacenza, Titania Pasqualini, Silvia Gottlieb, Pablo Knoblovits, Pablo R. Costanzo, Jorge Stewart Usher, Rodolfo A. Rey, María P. Martínez, Sergio Aszpis

**Affiliations:** ^1^Servicio de Endocrinología y Metabolismo, Unidad Asistencial “Dr. César Milstein”, Buenos Aires, Argentina; ^2^Sección Endocrinología, Crecimiento y Desarrollo, Departamento de Pediatría, Hospital Italiano de Buenos Aires, Buenos Aires, Argentina; ^3^División de Endocrinología, Hospital de Niños Dr. Ricardo Gutiérrez and, Centro de Investigaciones Endocrinológicas (CEDIE, CONICET), Buenos Aires, Argentina; ^4^Servicio de Endocrinología, Metabolismo y Medicina Nuclear, Hospital Italiano de Buenos Aires, Buenos Aires, Argentina; ^5^Consultorio de Endocrinología, Centro Médico Haedo, Haedo, Argentina; ^6^Sección Andrología, División Endocrinología, Hospital Durand, Buenos Aires, Argentina

## Abstract

The aim of the study was to establish the characteristics of presentation of 94 patients with Kinelfelter's syndrome (KS) referred to the endocrinologist at different ages. The diagnosis of KS was more frequent in the age group between 11 and 20 years (46.8%). Most of the patients (83.7%) showed the classic 47,XXY karyotype and 7.1% showed a 47,XXY/46,XY mosaicism. Half of the patients younger than 18 years presented mild neurodevelopmental disorders. The most frequent clinical findings were cryptorchidism in prepubertal patients, and small testes, cryptorchidism, and gynecomastia in pubertal patients. FSH, LH, AMH, and inhibin B levels were normal in prepubertal patients and became abnormal from midpuberty. Most adults were referred for small testes, infertility, and gynecomastia; 43.6% had sexual dysfunction. Testosterone levels were low in 45%. Mean stature was above the 50th percentile, and 62.5% had BMI ≥25.0 kg/m^2^. In conclusion, the diagnosis of Klinefelter syndrome seems to be made earlier nowadays probably because pediatricians are more aware that boys and adolescents with neuro-developmental disorders and cryptorchidism are at increased risk. The increasing use of prenatal diagnosis has also decreased the mean age at diagnosis and allowed to get insight into the evolution of previously undiagnosed cases, which probably represent the mildest forms. In adults average height and weight are slightly higher than those in the normal population. Bone mineral density is mildly affected, more at the spine than at the femoral neck level, in less than half of cases.

## 1. Introduction

The original clinical presentation of Klinefelter's syndrome was described by Klinefelter's, Reifenstein, and Albright in 1942, who reported 9 adult males with gynecomastia, small and firm testes, azoospermia, and elevation of serum FSH with functional Leydig, cells [1]. In 1959, using cytogenetics, Jacobs and Strong described the existence of an extra X chromosome, that is, 47,XXY karyotype, in a male with Klinefelter's syndrome [2]. This classical karyotype is present in approximately 90% of the cases. Patients with mosaicisms or with pure cell lineages carrying one or more extra X chromosomes and at least one Y chromosome (e.g., 47,XXY/46,XY, 47,XXY/45,X/46,XY, 48,XXXY, 49,XXXXY, 48,XXYY, etc.) are also considered as Klinefelter's syndrome variants, although the clinical presentation may be quite different in some cases [3].

Because chromosomal and gonadal features are atypical in Klinefelter's syndrome, the condition has been included as a disorder of sex development by the International Consensus Conference organized by the Lawson Wilkins Pediatric Endocrine Society and the European Society for Paediatric Endocrinology in 2006 [4, 5]. Furthermore, Klinefelter's syndrome is characterized by a genetic, whole gonadal dysfunction [6], affecting germ cells from early fetal life [7, 8] and Sertoli's and Leydig's cells from mid-puberty [9].

From an endocrinological standpoint, patient's complaints may vary, according to gonadal dysfunction, from signs of sex hormone deficiency in young adults to infertility in a male without other signs of hypogonadism. Before puberty, the condition is usually underdiagnosed due to the fact that childhood is a period characterized by physiologically low androgen levels and no sperm production [3]. Cryptorchidism and mild developmental disorders may be the alerting signs.

The main objective of this study was to establish the frequency and clinical characteristics of the different forms of presentation of Klinefelter's syndrome at different ages in a large cohort of patients.

## 2. Patients and Methods

We performed a retrospective chart analysis that included all patients with a confirmed diagnosis of Klinefelter's syndrome of 5 different centers in Buenos Aires, Argentina, from 1982 to 2008. The definition of Klinefelter's syndrome, as inclusion criterion for this study, required the availability of a karyotype consisting of an X-chromosome polysomy and at least one Y chromosome, either as a single lineage or as a mosaicism. The karyotype was analyzed in at least 30 metaphases.

The study was approved by the review boards of the participating institutions.

The main outcome measures were age at diagnosis, frequency of history or existence of cryptorchidism, gynecomastia, neuro-developmental disorders and signs of hypogonadism and serum hormone levels (FSH, LH, testosterone, AMH, inhibin B, estradiol, prolactin, TSH, and thyroid hormones), lipid and carbohydrate profiles, sperm analysis, and bone mineral density measurement at referral. Results were expressed as the prevalence (frequency of presentation at referral) or as the mean ± standard deviation or median and range, as applicable. Where necessary, quantitative variables were transformed into qualitative ones. For instance, hormone levels were classified as normal, elevated, or low according to the reference ranges for age. Data were analyzed using Instat Statistical Software (GraphPad Software, Version 3.01). Pearson's correlation coefficients and two-sample *t*-test for continuous variables were used. *P* values below 0.05 were considered to indicate statistical significance.

## 3. Results

**Figure 1:**
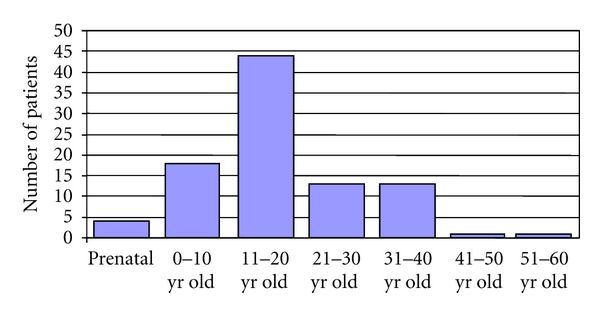
Age at diagnosis of the 94 patients of the present study.

Records from 98 patients with Klinefelter's syndrome were analyzed: 44 of them were under 18 yr old at referral (18 were under 10 yr old and 26 were between 10 and 17.9 yr old), and 54 were aged 18 yr or older. The most prevalent age range at diagnosis was 11–20 yr (Figure 1). A prenatal diagnosis was made in 4 cases. Karyotype was 47,XXY in 83.7% and 47,XXY/46,XY in 7.1% of the 98 patients; the other variants were homogeneously infrequent (Table 1).

### 3.1. Pediatric and Adolescent Patients with Klinefelter's Syndrome

The age group below 18 yr old was subclassified according to their pubertal status in prepubertal (median age 5.07 yr, range 0.75–9.28 yr) and pubertal (median age 14.3 yr, range 10.1–17.7 yr). In the prepubertal patients, cryptorchidism and neuro-developmental disorders, including behavior and/or learning difficulties, were the most prevalent findings (Table 2). In pubertal patients, the most frequent findings were small testes, neuro-developmental disorders, and gynecomastia.

Anthropometric evaluation was performed in boys and adolescents. The mean ± SD height standard deviation score (SDS) was 0.3 ± 1.8 (median 0.7, range −4.1 to 3.6) in prepubertal patients, and 1.1 ± 1.1 (median 1.1, range −1.4 to 2.9) in pubertal patients. SDS 0 represents the 50th percentile for the Argentine population, −2 is the 3rd percentile, and +2 is the 97th percentile. Our data indicate that pubertal patients with Klinefelter's syndrome were significantly higher (*P* < 0.0001). While height SDS above +2 was found in one prepubertal patient, it was above +2 in 5 pubertal patients. There was a significant correlation between height SDS and age (*n* = 40, *r* = 0.304, *P* < 0.05). Weight for height was adequate in most patients, except one prepubertal and 7 pubertal boys, with a body mass index (BMI) above the 95th percentile for age. Evaluation of the genitalia showed the existence of reduced penile size in 16.7% and 11.5% of prepubertal and pubertal patients, respectively, (Table 2). Testes were bilaterally nonpalpable in 22.2% of prepubertal patients. In those with palpable gonads, testicular volume was 1-2 mL in all cases except three (21.4%), who had abnormally small testes (<1 mL). In pubertal patients, testicular volume was below normal in all patients in the Tanner stages 3–5 (median 3.7 mL, range 1–8 mL). Bilateral gynecomastia was observed in 11 cases (42.3%). Varicocele was found in 3 cases (11.5%).

**Figure 2:**
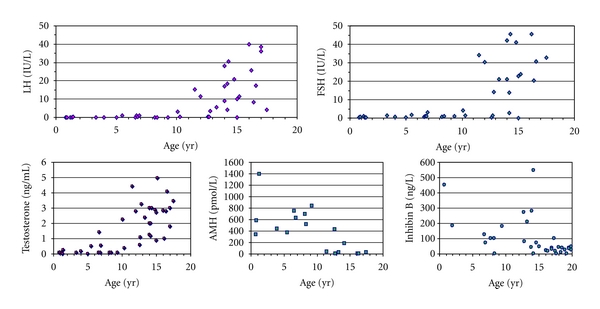
Serum hormone levels in pediatric and adolescent patients with Klinefelter's syndrome.

Evaluation of the reproductive axis hormone levels was available during followup in 13 prepubertal and 28 pubertal patients with Klinefelter's syndrome (Figure 2). FSH, LH, testosterone, inhibin B, and AMH levels were normal in all prepubertal boys. In pubertal patients, FSH and LH increased above the normal at 12.8 yr and 13.8 yr, respectively. A negative correlation was found between age and FSH (*r* = −0.62,  *P* < 0.001) and age and LH (*r* = −0.59, *P* < 0.001). Serum AMH and inhibin B fell below normal from pubertal Tanner stage 3. Testosterone was below normal, and hormone replacement therapy was indicated, in 7 of 28 pubertal patients (25%) before the age of 18 yr.

### 3.2. Adult Patients with Klinefelter's Syndrome

Infertility and small testes were the most prevalent complaints in adult patients (Table 3). Sexual function inquiry revealed reduced libido in 27.5% of patients and erectile dysfunction in 29.3% (Table 3). Information on maximal education level was available in 26 patients: 42.3% attained tertiary education.

Anthropometric evaluation revealed a median height above the 50th percentile for the Argentine male population (mean ± SD = 178.8 ± 9.0 cm, median 180 cm, range 161–200 cm) and a slight overweight (mean ± SD weight 83.6 ± 21.0 kg, median 83.5 kg, range 53–171; mean ± SD BMI 26.4 ± 5.5 kg/m^2^, median 25.9 kg/m^2^, range 17.2–51.1 kg/m^2^) 19 patients (47.5%) had a BMI between 25 and 29.9 kg/m^2^, and 6 patients (15%) were overtly obese (BMI  > 30 kg/m^2^). Eunuchoid proportions were observed in 35.2% of patients.

Evaluation of the genitalia showed small testes in all cases (median 3.5 mL, range 1–8 mL), varicocele in 23.3%, and gynecomastia in 31.3% (bilateral in most of the cases).

**Figure 3:**
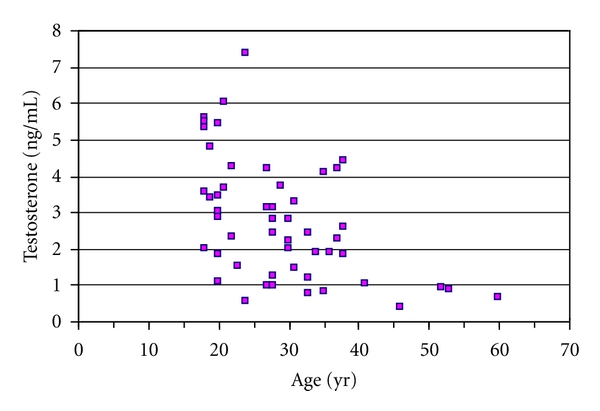
Serum testosterone levels in adult patients with Klinefelter's syndrome. Age versus testosterone: *r* : −0.5, *P* < 0.001.

Laboratory measurements (Table 4) revealed elevated FSH in 100% of cases and LH in 83%. Half of the patients with normal LH had subnormal total testosterone levels. Total testosterone decreased with age (Figure 3), and a negative correlation (*r* = −0.50,*P* < 0.001) was found between these variables. In the adult group as a whole, total testosterone was below normal in 45.1% of the cases. Prolactin was slightly elevated in 33% and estradiol in 6.2% of the cases. Semen analysis showed azoospermia in 89.3% of the cases and oligozoospermia in the remaining cases. No association was found with the karyotype. Total cholesterol was >200 mg/dL in 40%, LDL was >130 mg/dL in 50%, and HDL was <45 mg/dL in 47.4% of the cases. Fasting glucose levels were within the normal range in all patients.

Evaluation of bone mineral density (BMD) by DXA was performed in 27 adult patients. Results of BMD at lumbar spine were normal in 59.3% and showed osteopenia in 33.3% and osteoporosis in 7.4% of the cases. At the femoral neck level, BMD was normal in 72% and showed osteopenia in 28% of the cases, with no cases of osteoporosis.

## 4. Discussion

Klinefelter's syndrome is the commonest chromosomal abnormality in humans, with an estimated prevalence of 1 in 600 live births in males [3]. However, the condition is usually underdiagnosed, with an estimated 25% of the expected number of patients being ever diagnosed, and only a minority being diagnosed in childhood [10]. This may be due to the fact that Klinefelter's syndrome was initially a clinical diagnosis of adult males classically described as tall, with gynecomastia, small testes, azoospermia, sparse body hair, narrow shoulders, and broad hips. Those XXY males not presenting these typical features are probably missed, as are almost 90% of prepubertal patients in whom the diagnosis of hypogonadism is usually delayed until puberty [6]. Here we report the clinical and biochemical findings in a large cohort of Klinefelter's patients from pediatric age through adulthood. This is the largest series of patients with Klinefelter's syndrome in the literature. We are aware that a selection bias exists in our series, since all patients had been referred to a pediatric or adult endocrinologist. Therefore, the prevalence of clinical and biochemical findings in children, adolescents, and adults with Klinefelter's syndrome we report herein is applicable to those expected in an endocrinology setting.

The most prevalent age at diagnosis in our series was during adolescence, with more than half of our patients being diagnosed before adult age. This may reflect the fact that pediatricians and pediatric neurologists are increasingly aware of the increased prevalence of XXY karyotype in boys with mild neuro-developmental disorders, resulting in a rise in the diagnosis and early referral to the pediatric endocrinologist. Another reason may be the fact that cryptorchidism, a frequent cause of referral to the pediatric endocrinologist, is more frequent in patients with Klinefelter's syndrome [11–14]. In fact, the frequency of cryptorchidism was high in our series, mainly in the pediatric population. The almost tenfold higher prevalence of Klinefelter's syndrome in cryptorchid boys [11, 12] supports the indication of a karyotype analysis.

In coincidence with the initial characterization of long-term outcomes [15] and, more recently, of longitudinal growth in patients with Klinefelter's syndrome [16], our patients were slightly taller than the general male population. In boys and adolescents, we observed a positive correlation between age and stature. Eunuchoid proportions cannot be ascribed to hypoandrogenism since the increased height is present before pubertal onset, but rather to the existence of an extra copy of the SHOX gene, mapping to X and Y chromosomes and involved in linear growth [17]. It should be noted, however, that the height of 19% of our adult patients fell below the 50th percentile for the Argentine population, indicating that tall stature is not a prerequisite for the diagnosis of Klinefelter's syndrome.

A dissociated gonadal dysfunction with lack of sperm in semen and reduced testis volume, reflecting a severe disorder of the seminiferous tubules, with normal/low testosterone levels, indicating a mild dysfunction of the interstitial compartment, was described in the initial report by Klinefelter's and colleagues [1]. Our results, together with other recent reports [7–9, 18, 19], further indicate that the establishment of the dysfunction of the seminiferous tubule components is also dissociated. In fact, germ cells are already affected in early postnatal life while Sertoli's cells secrete normal levels of AMH and inhibin B until mid-puberty. Similarly, Leydig's cells seem to produce androgens normally until mid-puberty; thereafter, although androgen levels might be within the normal range in a proportion of patients with Klinefelter's syndrome, there is an increase in LH, indicating a suboptimal Leydig cell functional capacity. Our results show that FSH levels are increased approximately one year before LH levels during puberty in patients with Klinefelter's syndrome, which suggests that Sertoli's cell function is affected not only more severely than that of Leydig's cells but also earlier. The age for testosterone replacement in these patients is controversial, yet clear clinical and biochemical signs of hypoandrogenism are an undisputed indication for the initiation of androgen therapy [20]. In our series, 7 patients required testosterone replacement before the age of 18 yr. Decreased Leydig's cell function has also been detected by measuring INSL3 [21, 22] and confirmed by histological studies showing increased interstitial fibrosis and decreased androgen receptor expression with age [7, 23]. Nonetheless, the indication for androgen therapy should not be unnecessarily anticipated, since the likelihood of obtaining sperm seems to decrease once treatment is initiated [24].

Lipid metabolism was abnormal in a variable proportion of our patients, as revealed mainly by the measurement of total cholesterol and its fractions, in coincidence with a recent report [25]. The latter study shows that the disorder in the lipid metabolism could not be modified by androgen therapy. Bone mineral density was mildly decreased in our patients, in line with a recent report in Korean males, which showed no significant correlation with androgen deficiency [26].

In conclusion, the diagnosis of Klinefelter's syndrome was more frequent between 11 and 20 yr of age in our series, probably due to the fact that in the last years pediatricians have become more aware of the likelihood of the diagnosis in boys and adolescents with neuro-developmental disorders, cryptorchidism, and small testes. The increasing use of prenatal diagnosis has also decreased the mean age at diagnosis and allowed to get insight into the evolution of previously undiagnosed cases, which probably represent the mildest forms. The function of the hypothalamic-pituitary-gonadal axis is preserved during infancy and childhood, with a progressive decline of the seminiferous tubule compartment from mid-puberty. The Leydig cell compartment shows a mild dysfunction compensated by an increase in LH during late puberty and early adulthood. As previously described, hypoandrogenism occurs in an increasing proportion of patients with age. Small testes, infertility, gynecomastia. decreased libido, and sexual or ejaculatory dysfunction were associated complaints. In adults average height and weight are slightly higher than in the normal population. Bone mineral density is mildly affected, more at the spine than at the femoral neck level, in less than half of cases.

## Figures and Tables

**Table 1:** Karyotypes found in the 98 patients of this study.

Karyotype	*n*	%
*Pure lineage*	*86*	*87.76*
47,XXY	82	83.67
48,XXXY	1	1.02
48,XXYY	3	3.06
*Mosaicisms*	*12*	*12.24*
47,XXY/46,XY	7	7.14
47,XXY/48,XXYY	2	2.04
47,XXY[4]/48,XXXY[2]/46,XY[44]	1	1.02
49,XXXXY[44]/48,XXXY[6]	1	1.02
47,XXY[36]/48,XXXY[1]/46,XX[1]/46,XY[2]	1	1.02

**Table 2:** Prevalence of clinical signs in pediatric and adolescent patients with Klinefelter's syndrome.

	Prepubertal (*n* = 18)	Pubertal (*n* = 26)
Neuro-developmental disorders	8 (44.4%)	14 (53.8%)
Small testes	3 (16.7%)	20 (76.9%)
Cryptorchidism	10 (55.5%)	6 (23.0%)
Gynecomastia	0 (0%)	11 (42.3%)
Small penis	3 (16.7%)	3 (11.5%)
Prenatal diagnosis	4 (22.2%)	0 (0%)
Pie bot	1 (5.5%)	0 (0%)
Varicocele	3 (11.5%)	3 (11.5%)
Dysmorphisms	1 (3.8%)	1 (3.8%)

**Table 3:** Prevalence of main complaints and clinical signs and symptoms in adult patients with Klinefelter's syndrome.

Main complaints	%
Infertility	34.8
Small testes	34.8
Gynecomastia	10.9
Erectile dysfunction	6.5
Hypergonadotrophic hypogonadism	4.3
Eunuchoid proportions	2.2
Decreased libido	2.2

Signs and symptoms	%

Small testes	100
Infertility	100
Eunuchoid proportions	35.2
Gynecomastia	31.3
Erectile dysfunction	29.3
Reduced libido	27.5
Varicocele	23.3
Neurodevelopmental disorders	22.0
Small penis	0

**Table 4:** Serum levels of reproductive axis hormones and carbohydrate and lipid profiles in adult patients with Klinefelter's syndrome.

	Mean ± SD	% Abnormal	Reference levels
FSH (IU/L)	35.4 ± 16.2	100	1–8
LH (IU/L)	22.3 ± 11.6	83.0	2–12
Total testosterone (ng/mL)	2.74 ± 1.65	45.1	2.8–8.8
Estradiol (pg/mL)	26.5 ± 13.2	6.3	18–44
Prolactin (ng/mL)	16.5 ± 11.3	33.3	5–20
TSH (*μ*U/mL)	2.11 ± 0.99	0.0	0.47–4.64
Fasting glucose (mg/dL)	91.5 ± 13.1	0.0	70–110
Total cholesterol (mg/dL)	189 ± 40	40.0	110–200
LDL (mg/dL)	124 ± 36	50.0	<130
HDL (mg/dL)	43 ± 7	47.4	>40
Triglycerides (mg/dL)	115 ± 77	15.0	50–200
